# Urinary Biomarkers at Early ADPKD Disease Stage

**DOI:** 10.1371/journal.pone.0123555

**Published:** 2015-04-13

**Authors:** Katja Petzold, Diane Poster, Fabienne Krauer, Katharina Spanaus, Gustav Andreisek, Thi Dan Linh Nguyen-Kim, Ivana Pavik, Thien Anh Ho, Andreas L. Serra, Laura Rotar

**Affiliations:** 1 Institute of Physiology and Zurich Center for Integrative Human Physiology, Zurich, Switzerland; 2 Division of Nephrology, University Hospital Zurich, Zurich, Switzerland; 3 Institute of Clinical Chemistry, University and University Hospital Zurich, Zurich, Switzerland; 4 Department of Diagnostic and Interventional Radiology, University Hospital Zurich, Zurich, Switzerland; 5 Epidemiology, Biostatistics and Prevention Institute, University of Zurich, Zurich, Switzerland; 6 EuroCYST Initiative, Coordination Center, University of Zurich, Zurich, Switzerland; 7 Division of Nephrology, Cliniques Universitaires Saint-Luc, Université catholique de Louvain Medical School, Brussels, Belgium; 8 Trancyst FP7-PEOPLE-MCA-ITN no. 317246, University of Zurich, Zurich, Switzerland; The University of Tokyo, JAPAN

## Abstract

**Background:**

Autosomal dominant polycystic kidney disease (ADPKD) is characterized by a decline in renal function at late disease stage when the majority of functional renal parenchyma is replaced by cystic tissue. Thus, kidney function, assessed by estimated glomerular filtration rate (eGFR) does not well represent disease burden in early disease. Here, we investigated various urinary markers for tubular injury and their association with disease burden in ADPKD patients at early disease course.

**Methods:**

ADPKD patients between 18 and 40 years with an eGFR greater or equal to 70 ml per min per 1.73m^2^ were eligible for this cross-sectional study. Urinary Neutrophil Gelatinase-Associated Lipocalin (NGAL), Kidney Injury Molecule-1 (KIM-1), and Uromodulin (UMOD) were investigated by Enzyme-Linked Immunosorbent Assay. Clara Cell Protein 16 (CC16) was investigated by Latex Immuno Assay. Cryoscopy was performed to assess urine osmolality and Urinary Albumin-to-Creatinine Ratio (UACR) was calculated. The association and the predictive properties of the markers on eGFR and height adjusted total kidney volume (htTKV) was evaluated using multiple regression analysis, incorporating different control variables for adjustment. Internal bootstrapping validated the obtained results.

**Results:**

In 139 ADPKD patients (age 31 ±7 years, mean eGFR of 93 ± 19 ml per min per 1.73 m^2^) the total kidney volume was negatively correlated with eGFR and UMOD and positive associated with age, UACR, KIM-1 and urine osmolality after adjustment for possible confounders. Urine osmolality and htTKV were also associated with eGFR, whereas no association of CC16, NGAL and UMOD with eGFR or htTKV was found.

**Conclusion:**

UACR and urinary KIM-1 are independently associated with kidney size but not with renal function in our study population. Urine osmolality was associated with eGFR and kidney volume following adjustment for multiple confounders. Despite statistical significance, the clinical value of our results is not yet conceivable. Further studies are needed to evaluate the property of the aforementioned biomarkers to assess disease state at early ADPKD stage.

## Introduction

Autosomal dominant polycystic kidney disease (ADPKD) is one of the most common inherited kidney diseases. It is characterized by a decline of glomerular filtration rate at advanced disease stage and a high inter- and intrafamilial variability in age of end stage renal disease (ESRD) onset, implying a challenge to predict individuals’ disease progression. The development and continued accretion of cysts, as the most prominent feature in ADPKD, leads to a massive enlargement of the kidney and, subsequently, to a loss of its function. So far, no disease modifying treatment is available, except the recent approval of the vasopressin V_2_ receptor antagonist Tolvaptan in Japan. Due to glomerular hyperfiltration of the remaining nephrons kidney function stays stable over decades. Thus, traditional markers for kidney function like serum creatinine and estimated glomerular filtration rate (eGFR) have limited ability to accurately assess disease state and to predict progression in the early course of ADPKD. Increasing evidence suggests that total kidney volume qualifies as a marker for disease progression in ADPKD [1]. In fact, the disease state may be reflected more accurately by total kidney volume and kidney growth rate than renal functional parameters like eGFR or creatinine clearance [2]. Total kidney volume can be accurately assessed by Magnetic Resonance Imaging (MRI). However MRI derived kidney volume measurements are time and cost intensive, require high technical expertise and are not routine clinical practice.

Renal cystogenesis in ADPKD is a complex process, characterized by abnormalities in tubular cell proliferation, fluid secretion, extracellular matrix formation, and cell polarity [3]. The process results in an impaired filtration barrier, diminished tubular reabsorption, upregulation of tubular proteins and release of markers by recruited inflammatory cells, which can be detected in patients’ urine [4]. Such markers should have the property to define the patients’ state in a certain disease condition, to predict prognosis, and/or to quantify the effect of a pharmacological approach. Mayeux et al defined type 0 biomarkers (diagnostic biomarkers) reflecting the natural history and correlating with clinical indices and biomarkers of type 1 (predictive biomarkers) capturing the effect of an intervention [5,6]. Biomarkers of type 0 that reflect tubular damage and have been under investigation in various settings of kidney disease are Neutrophil Gelatinase Associated Lipocalin (NGAL), Kidney Injury Molecule-1 (KIM-1), Uromodulin (UMOD), Clara Cell Protein 16 (CC16) and albuminuria [6]. NGAL has been extensively investigated as a biomarker, due to its rapid increase in different settings like acute kidney injury, cardiac surgery, and kidney transplantation [7–11]. KIM-1 does not occur in human urine under physiological conditions and has been described as progression marker in kidney disease [2]. UMOD, the most abundant protein in human urine, regulates tubular function and shows protective properties against uropathogenic Escherichia coli and nephrolithiasis [12]. Decreasing levels of urinary UMOD have been reported in various settings of chronic kidney disease (CKD), like glomerulonephritis, diabetic nephropathy or tubulointerstitial nephropathy [12–15]. Urinary CC16 is consistently associated with defective endocytic uptake by the proximal tubule. CC16 levels are increased in patients with diabetic and HIV-induced nephropathy, as well as in renal Fanconi syndrome [16,17]. There is an unmet need to discover new biomarkers that allow an easy and non-invasive assessment of ADPKD disease state. Here, we investigated the potential properties of the aforementioned markers for assessing disease state by evaluating their association with kidney volume and function in patients at early stage of ADPKD.

## Methods

### Study Subjects

Subjects of the well-described SUISSE ADPKD cohort were eligible for enrolment [18,19]. Male and female patients with proven ADPKD diagnosis, examined by kidney ultrasonography, according to Ravine criteria, and a positive family history were eligible when aged between 18 and 40 and presenting with an eGFR greater or equal to 70 ml per min per 1.73m^2^ as shown in Table 1 [20]. A proof of a mutation in the *PKD1* or *PKD2* genes was required for enrolment of patients without family history (sequencing analysis by Athena Diagnostics Inc., Worcester, MA, USA). The study was conducted according to the Declaration of Helsinki and Good Clinical Practice Guidelines and was approved by the local ethical board. All patients provided written informed consent.

**Table 1:** Eligibility criteria^19^.

• Age 18 to 40
• GFR ≥ 70 ml per min per 1.73m^2^ (Cockcroft-Gault formula)
■ Clinical diagnosis of ADPKD based on kidney imaging (modified Ravine criteria) and family history
■ Positive family history for ADPKD
○ patients < 30 years: ≥ 2 cysts in either kidney
○ patients ≥ 30 years: ≥ 2 cysts in each kidney
• Negative family history for ADPKD cystic kidney disease by sonography: proof of a mutation in the PKD1 or PKD2 gene
• Patient provided written informed consent

### Study Procedure

Subjects were invited to the outpatient clinic at the Division of Nephrology (University Hospital Zurich). At study visit the medical history was obtained, including medication and ADPKD related complications. Blood pressure measurement was done in duplicate at each arm after 5 minutes of rest in sitting position using an oscillometric blood pressure device (Boso-Medicus, Jungingen, Germany). Hypertension was defined as systolic blood pressure above 140 mmHg and/or diastolic blood pressure above 90 mmHg or antihypertensive treatment. Fasting spot urine samples were collected after voiding the first urine of the day to measure creatinine beside the potential biomarkers. Blood samples were centrifuged and aliquoted, according to a standardized process, to obtain serum. Serum and spot urine aliquots were stored at—80°C before analysis.

### Laboratory Analysis

At study visit, serum creatinine was measured according to modified Jaffé method traceable to an isotope-dilution mass spectroscopy reference. Estimated GFR was calculated by applying the CKD-EPI equation [21]. NGAL (BioPorto Diagnostics A/S, Hellerup, Denmark) and KIM-1 (R&D Systems Inc., Abingdon, UK) were analyzed using commercially available Enzyme-linked Immunosorbent Assays (ELISA) according to manufacturers protocol. UMOD was analyzed by a well established ELISA based on a sheep anti-human uromodulin antibody (K90071C; Meridian Life Science, Memphis, TN) as the capture antibody, a mouse monoclonal anti-human Tamm–Horsfall protein antibody (CL 1032A; Cedarlane Laboratories, Burlington, NC) as the primary antibody, and a goat anti-mouse IgG (H+L) horseradish peroxidase–conjugated protein (172.1011; Bio-Rad Laboratories, Inc., Hercules, CA) as a secondary antibody. Human uromodulin (AG 733, stock solution: 100 *μ*g/ml; EMD Millipore, Temecula, CA) was used to establish the standard curve [22]. CC16 was analyzed with continuous flow Latex Immuno Assay (LIA) and an assayable concentration of CC16 between 0.3 and 40 μg/L [23]. Albuminuria was assessed using Synchron Systems for Microalbumin (Beckman Coulter Inc., Brea, California, USA). The urinary albumin-to-creatinine ratio (UACR) was calculated as follows: Albumin (mg/dl) x 1/creatinine (mg/dl) x 1000 μg/mg. The analysis of urine osmolality was performed by cryoscopy using a freezing point depression Advanced 2020-BIO Multi-Sample Osmometer (Advanced Instruments Inc., Norwood, Massachusetts, USA). All samples were handled in a uniform way and underwent no freeze-thaw cycle before analyzed in duplicate.

### Magnetic Resonance Imaging

Patients underwent magnetic kidney imaging without contrast media according to a standardized imaging protocol. The imaging was performed using a Signa Excite HDx system (GE Healthcare, Waukesha, WI, USA) and signal perception was obtained with an eight-channel antero-posterior-phased array surface coil. Trans-axial sequences consisted of two breathhold T1-weighted fast-spoiled gradient echo sequences with 3 and 4 mm slice thicknesses. Additionally, a trans-axial T2-weighted fast spin echo sequence with respiratory triggering was performed with 3 mm slice thickness. Right and left kidney volumes were measured and calculated using the Advantage Windows workstation (4.4 GE Healthcare, Buc, France). Total kidney volume (TKV) was calculated by adding the volume of the left and right kidney. Measurements of renal volume were done in a blinded way by two trained and independent observers. The renal hilum and the vessels were excluded from renal volume calculation. Variability was calculated as concordance correlation coefficients (95% CI) and were 1.000 (0.999–1.000) for intraobserver and 0.996 (0.995–0.999) for interobserver correlations [24].

### Statistical Analysis

SAS 9.4 was used for data analysis. A plausibility check of the data preceded the statistical analysis. Univariate methods were used to characterize study population. Values are given in means with standard deviation. TKV and height adjusted kidney volume (htTKV) are reported as median because of a skewed distribution of these parameters. Median is reported with interquartile range.

Spearman’s correlation coefficient (r) was calculated to describe the correlation between biomarkers and eGFR and htTKV. To calculate r, reflecting the relative variance part, all values of the parameters are sorted and given a rank [25]. A positive r indicates a concordant association, whereas a negative r stands for an opposing association.

The association of biomarkers with eGFR and htTKV was evaluated by incorporating different control variables for adjustment and following a multiple linear regression approach. A stratum specific correlation analysis was performed for binary and ordinal variables. Multiple regression analysis gives information about importance and size effect of the predictors on the response variable, and about the interaction between predictors. Prerequisites, like the independence of predictors to each other, were evaluated and fulfilled. The models were determined with scatterplots and correlation analysis to verify linearity between outcome variable and predictors. Subsequently the regression equation was formed. Adjusted R^2^ and p-value were calculated as statistic measures. R^2^, the coefficient of determination, reflects the proportion response variation that is explained by the predictors [26]. Predictor variables were selected according to logical considerations and added sequentially to the models. The models were compared using the Akaike information criterion (AIC). The AIC is based on the likelihood and determines which model is more likely to be correct and comes closer to the “truth” [27]. The smaller the AIC value the more realistic the model is, assuming, that a robust model predicts the data well containing preferably a low number of predictors meeting the requirement for parsimony and avoiding overfitting [28]. Bootstrapping was applied to estimate the 2.5^th^ and 97.5^th^ percentile confidence intervals for each model. Our dataset was used as a pool from which 500 new datasets of the same size were randomly drawn with replacement to internally validate our model results.

## Results

### Demographics

Between April 2006 and April 2011 139 ADPKD patients were consecutively enrolled in the study. The mean age was 31±7 years and 85 (61%) patients were male. Hypertension was present in 82 (78%) patients, and 80 (58%) patients received antihypertensive medication. Among those, 50 patients received angiotensin-converting enzyme (ACE) inhibitors and angiotensin receptor blockers (ARBs), 16 received diuretics and 14 were treated with calcium antagonists. The mean eGFR was 93±19 ml per min per 1.73 m^2^ and 74 (53%) of the patients had an eGFR greater than 90 ml per min per 1.73 m^2^. The median TKV was 860 cm^3^ (IQR, 568 to 1191 cm^3^) and 52 (37%) patients had a TKV greater than 1000 cm^3^. The median htTKV was 455 cm^3^ (IQR, 17 to 669 cm^3^). The mean body mass index (BMI) was 24±4 kg per m^2^, 5 (4%) patients had a BMI lower than 18.5 kg per m^2^ and 53 (38%) patients a BMI above 25 kg per m^2^ (Table 2).

**Table 2:** Characteristics of study cohort.

	ADPKD
	n = 139
**Age**—years	31±7
**Sex**—no. (%)	
Female	54 (39)
Male	85 (61)
**Body mass index**—kg per m^2^	24±4
BMI <18.5	5 (4)
BMI 18.5–25	81 (58)
BMI >25	53 (38)
**eGFR**—ml per min per 1.73m^2^	93±19
CKD stage 1	74 (53)
CKD stage 2	61 (44)
CKD stage 3	4 (3)
**TKV**—cm^3^	860 (568 to 1191)
**htTKV**—cm^3^ per m	455 (317 to 669)
**Hypertension—**no. (%)*	
Yes	82 (78)
No	23 (22)
**Blood pressure**—mmHg	
Systolic	131±16
Diastolic	83±11
**Antihypertensive Medication**—no. (%)	
ACE / ARB	50 (36)
Calcium antagonist	14 (10)
Diuretics	16 (12)

Abbreviations: eGFR—estimated glomerular filtration rate. TKV—total kidney volume, htTKV—height adjusted total kidney volume, ACE—angiontensin converting enzyme, ARB—angiotensin II receptor blocker. Values are means ± standard deviation and numbers (percentage), TKV and htTKV are reported as median (interquartile range)

*total number of observations = 105

### Analysis of biomarker

The results of the urinary parameters osmolality, NGAL, KIM-1, UMOD, UACR and CC16 were tabulated for the complete cohort and stratified for eGFR and TKV (Table 3). Osmolality was measured in 139 spot urine samples. All other potential biomarkers were measured in 132 samples. The median urinary osmolality was 364 mosmol per kg H_2_O (IQR, 257 to 533 mosmol per kg H_2_O). The median values were 9.8 μg per g creatinine (IQR, 5.3 to 23.7 μg per g creatinine) for NGAL, 274.6 ng per g creatinine (IQR, 131.3 to 457.3 ng per g creatinine) for KIM-1, and 16.3 mg per g creatinine (IQR, 10.2 to 26.7 mg per g creatinine) for UMOD. In the whole cohort, UACR was 14.0 mg per g creatinine (IQR, 8.4 to 23.1 mg per g creatinine) and the median for CC16 was 2.8 μg per l per g creatinine (IQR, 2.0 to 6.2 μg per l per g creatinine). The median of KIM-1 was significantly higher among patients with TKV above 1000 cm^3^ than among patients with TKV lower or equal 1000 cm^3^. Osmolality, NGAL, UMOD, UACR and CC16 were similar among patients with an eGFR above 90 ml per min per 1.73m^2^ and less or equal 90 ml per min per 1.73m^2^.

**Table 3:** Biomarker Analysis.

	n	Complete Cohort	eGFR >90 ml per min per 1.73m^2^	eGFR ≤90 ml per min per 1.73m^2^	TKV ≤1000 cm^3^	TKV >1000 cm^3^
**Osmolality**—mosmol per kg H_2_O	139	364 (257 to 533)	377 (266 to 534)	360 (243 to 506)	330 (236 to 497)	417 (332 to 547)
**NGAL**— μg per g_creatinine_	132	9.8 (5.3 to 23.7)	8.7 (4.7 to 22.8)	11.3 (5.8 to 24.3)	12.5 (5.3 to 24.3)	9.0 (5.3 to 21.3)
**KIM-1**—ng per g_creatinine_	132	274.6 (131.3 to 457.3)	272.0 (113.9 to 448.8)	274.6 (161.3 to 479.9)	211.6 (106.4 to 362.5)	391.3 (196.1 to 581.0)*
**UMOD**—mg per g_creatinine_	132	16.3 (10.2 to 26.7)	18.1 (10.9 to 29.8)	14.8 (9.1 to 25.6)	19.4 (10.7 to 28.5)	13.6 (8.3 to 21.6)
**UACR**—mg per g_creatinine_	132	14.0 (8.4 to 23.1)	11.6 (6.9 to 21.8)	15.4 (9.9 to 27.2)	12.0 (7.7 to 21.3)	15.2 (10.4 to 31.1)
**CC16** – μg per l per g_creatinine_	132	2.8 (2.0 to 6.2)	3.0 (2.0 to 5.9)	2.6 (2.0 to 6.2)	2.6 (2.0 to 4.8)	3.2 (1.7 to 8.9)

Abbreviations: NGAL—Neutrophil Gelatinase Associated Lipocalin, KIM-1—Kidney Injury Molecule-1, UMOD—Uromodulin, UACR—Urinary Albumin-to-Creatinine Ratio, CC16—Clara Cell Protein 16. Values are reported as median (interquartile range)

* *p* < 0.05

### Correlation of biomarker with indices of disease progression

**Fig 1:**
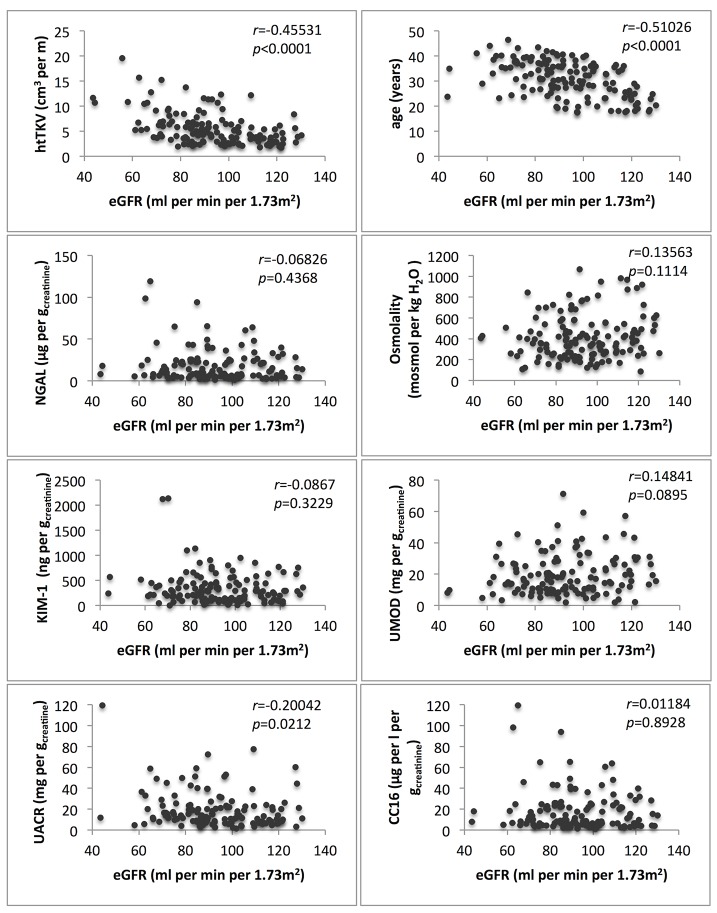
Estimated glomerular filtration rate (eGFR) and parameter distribution.

**Fig 2:**
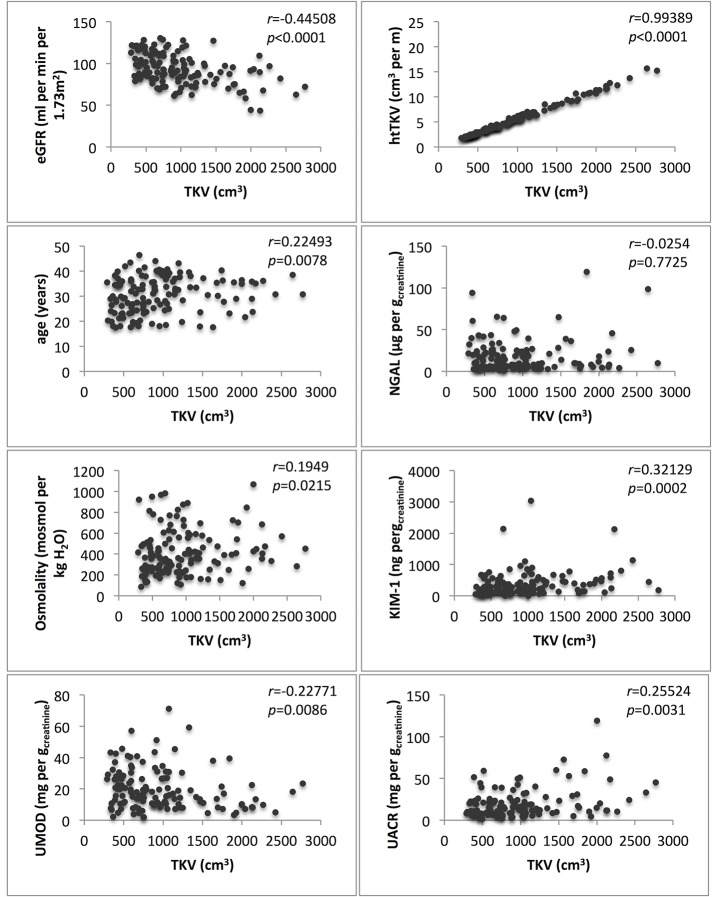
Total kidney volume (TKV) and parameter distribution.

Table 4 shows the correlation of biomarkers with eGFR and TKV. Estimated GFR was negatively correlated with TKV (r = -0.44508, p<0.05), htTKV (r = 0.45531, p<0.05), age (r = -0.51026, p<0.05) and UACR (r = -0.20042, p<0.05). TKV was negatively correlated with eGFR (r = -0.44508, p<0.05) and UMOD (r = -0.22771 p<0.05) and positively correlated with age (r = 0.22493, p<0.05), urinary albumin (r = 0.25524, p<0.05), osmolality (r = 0.1949, p<0.05) and KIM-1 (r = 0.32129, p<0.05) (Table 4). Biomarker distribution is shown in Figs 1 and 2.

**Table 4:** Spearman Correlation Coefficient *r*.

	eGFR	TKV	htTKV	Age	NGAL	OSMOL	KIM-1	UMOD	UACR	CC16
**eGFR**	1	-0.44508*	-0.45531*	-0.51026*	-0.06826	0.13563	-0.0867	0.14841	-0.20042*	0.01184
**TKV**	-0.44508*	1	0.99389*	0.22493*	-0.0254	0.1949*	0.32129*	-0.22771*	0.25524*	0.12924

Abbreviations: eGFR—estimated glomerular filtration rate, TKV—total kidney volume, htTKV—height adjusted total kidney volume, NGAL—Neutrophil Gelatinase Associated Lipocalin, OSMOL—Osmolality, UMOD—Uromodulin, KIM-1—Kidney Injury Molecule-1, UACR—Urinary Albumin-Creatinine-Ratio, CC16—Clara Cell Protein 16.

* *p* < 0.05

### Regression analysis for htTKV and eGFR as outcome parameter

Simple and multiple linear regression analysis were applied to delineate the independent associations of urinary biomarkers with eGFR and htTKV. Kidney volume is affected by a number of *a priori* known biological factors, e.g. age, gender, and glomerular filtration rate. Predictive variables were chosen in a predefined step-wise approach: In model 1 (Table 5) eGFR (β = -0.45968, p<.0001) was selected as a predictor; the term eGFR captures race, age and gender. The prognostic power of eGFR to predict htTKV is 20.6% (R^2^ = 0.2055) with an AIC of -199.6. Bootstrapping revealed a percentile confidence interval of 0.0987 and 0.3374.

**Table 5:** Simple linear regression height adjusted total kidney volume (transformed to log htTKV) Model 1.

variable	Parameter estimate b	*p*	Standardized estimate β	Standard Error	Adjusted R^2^	AIC
Intercept	2.20103	<.0001	0	0.1145	0.2055	-199.6
eGFR^1^ —ml per min per 1.73m^2^	-0.00007127	<.0001	-0.45968	0.00001181		

^1^square root transformed

The selection of osmolality and UACR to model 1 as independent variables increased the R^2^ to 0.3373 (percentile CI, 0.2306–0.4755), and the AIC to -210.7 (Table 6; Model 2). An increase of UACR (b = 0.20465, p<.0001) and urine osmolality (b = 0.32114, p<.0001) is independently of renal function, race, age, and gender associated with an increase in htTKV. All predictors of model 2 are independently predictors of htTKV at an alpha level of 0.1%. Estimated GFR has the most predictive power of all 3 variables in this model (β = -0.42435). The standardized estimate β was calculated to evaluate the predictors independently of their transformation and their level of measurement.

**Table 6:** Multiple linear regression height adjusted total kidney volume (transformed to log htTKV) Model 2.

variable	Parameter estimate b	*p*	Standardized estimate β	Standard Error	Adjusted R^2^	AIC
Intercept	-0.29424	0.5704	0	0.51711	0.3373	-210.7
eGFR^1^ – ml per min per 1.73m^2^	-0.00006612	<.0001	-0.42435	0.00001149		
Osmolality^2^ – mosmol per kg H_2_O	0.32114	<.0001	0.30774	0.07825		
UACR^2^ – mg per g_creatinine_	0.20465	<.0001	0.31058	0.04985		

^1^ square root transformed

^2^ log transformed

Adjusted R^2^ of model 3 was 0.3366 after selection of KIM-1, NGAL, UMOD, CC16 to eGFR, osmolality and UACR (Table 7). A percentile confidence interval of 0.2866 to 0.5278 was obtained in bootstrap validation. Out of the seven aforementioned variables, eGFR, osmolality, UACR and KIM-1 are major factors to predict htTKV. In model 3, the variable UACR is the second largest predictor variable with a β of 0.30403. Osmolality had a β of 0.21408, and KIM-1 a β of 0.18993. NGAL, UMOD and CC16 had low β-values and were minor determinants in the prognosis of htTKV. In model 3 osmolality, UACR and KIM-1 are positively correlated with kidney volume. The AIC of model 3 was -209.9. The additional selection of KIM-1, NGAL, UMOD and CC16 did not increase R^2^ and did not change AIC.

**Table 7:** Multiple linear regression height adjusted total kidney volume (transformed to log htTKV) Model 3.

variable	Parameter estimate b	*p*	Standardized estimate β	Standard Error	Adjusted R^2^	AIC
Intercept	0.00847	0.9895	0	0.64145	0.3366	-209.9
eGFR^1^ —ml per min per1.73m^2^	-0.00006259	<.0001	-0.40173	0.00001166		
Osmolality^2^ —mosmol per kg H_2_O	0.2234	0.0186	0.21408	0.09366		
UACR^2^ —mg per g_creatinine_	0.20033	0.001	0.30403	0.05951		
KIM-1^2^ —ng per g_creatinine_	0.09432	0.0191	0.18993	0.0475		
NGAL^2^ – μg per g_creatinine_	-0.05253	0.2709	-0.09574	0.0397		
UMOD^2^ —mg per g_creatinine_	-0.04606	0.4653	-0.06085	0.06289		
CC16^2^ – μg per l per g_creatinine_	-0.00504	0.9212	-0.00794	0.05087		

^1^ square root transformed

^2^ log transformed

Subsequently different models were established to predict eGFR. In model 1 (Table 8) htTKV and osmolality were added on *priory* knowledge to predict eGFR. Both variables were independently associated with eGFR, and htTKV (β = -0.49803) had a larger association with eGFR compared with osmolality (β = 0.22936). The adjusted R^2^ for this model is 0.2515 (percentile CI, 0.1588–0.3809) and thus approximately 25% of eGFR variation is explained by htTKV and osmolality.

**Table 8:** Multiple linear regression estimated glomerular filtration rate (square root transformed eGFR) Model 1.

variable	Parameter estimate b	*p*	Standardized estimate β	Standard Error	Adjusted R^2^	AIC
Intercept	4899.70827	0.1014	0	2970.99215	0.2515	2214.2
htTKV^1^ – cm^3^ per m	-3212.08387	<.0001	-0.49803	483.52856		
Osmolality^1^ – mosmol per kg H_2_O	1549.70316	0.0027	0.22936	506.54097		

^1^ log transformed

In model 2 (Table 9) the predicting parameters htTKV, osmolality and UACR account for 22.09% of eGFR variation with an adjusted R^2^ of 0.2209. A percentile confidence interval of 0.1319 to 0.3738 was obtained in bootstrap validation. The predictor htTKV has the largest impact on the outcome in this model (β = -0.48736) and osmolality showed the second largest value for standardized estimate (β = 0.21857). UACR has a comparably low β.

**Table 9:** Multiple linear regression estimated glomerular filtration rate (transformed to square root eGFR) Model 2.

Variable	Parameter estimate b	*p*	Standardized estimate β	Standard Error	Adjusted R^2^	AIC
Intercept	4899.8604	0.1681	0	3534.68607	0.2209	2104.3
htTKV^1^ – cm^3^ per m	-3127.98641	<.0001	-0.48736	543.58333		
Osmolality^1^ – mosmol per kg H_2_O	1463.87536	0.0098	0.21857	557.90345		
UACR^1^ – mg per g_creatinine_	100.97551	0.7824	0.02388	364.80352		

^1^ log transformed

In model 3 (Table 10) the parameters htTKV, osmolality, UACR, NGAL, KIM-1, UMOD and CC16 were entered. Height adjusted total kidney volume (β = -0.47261; p<.0001) and osmolality (β = -0.27024; p = 0.006) were independently associated with changes of eGFR. The additional selection of NGAL, KIM-1, UMOD and CC16 resulted in a stable R^2^ and AIC. Bootstrapping revealed a percentile confidence interval of 0.1674 to 0.4153.

**Table 10:** Multiple lineare regression estimated glomerular filtration rate (transformed to square root eGFR) Model 3.

Variable	Parameter estimate b	*p*	Standardized estimate β	Standard Error	Adjusted R^2^	AIC
Intercept	170.01158	0.9697	0	4465.37589	0.2195	2108.4
htTKV^1^ – cm^3^ per m	-3333.33092	<.0001	-0.47261	564.97273		
Osmolality^1^ – mosmol per kg H_2_O	1809.95369	0.006	0.27024	646.61622		
UACR^1^ – mg per g_creatinine_	42.27981	0.9224	0.01	432.9461		
NGAL^1^– μg per g_creatinine_	-136.7632	0.6812	-0.03884	332.06115		
KIM-1^1^ – ng per g_creatinine_	185.45311	0.5123	0.05818	282.16858		
UMOD^1^ – mg per g_creatinine_	798.52678	0.0675	0.16434	432.84451		
CC16^1^ – μg per l per g_creatinine_	-138.45145	0.6963	-0.03397	353.90411		

^**1**^ **log transformed**

## Discussion

The cystogenesis in ADPKD replaces functional renal parenchyma and leads to a loss of kidney function during patients’ lifespan. Since GFR, a traditional parameter of renal function, is not able to accurately assess disease state in the early disease course, the interest in establishing urinary biomarkers for ADPKD has increased. In this cross-sectional study, we investigated the potential biomarkers osmolality, UACR, NGAL, UMOD, CC16, KIM-1 at a single time point in spot urine samples of 139 ADPKD patients with preserved renal function. Robust statistical approach demonstrated that urinary KIM-1, urinary osmolality and UACR are independently associated with kidney volume in our cohort of ADPKD patients.

An increase in urinary KIM-1, that is only fractionally expressed under physiological conditions, reflects tubular damage in the proximal S3 tubule segment as shown in acute and chronic kidney injury [29]. In our study, KIM-1 showed the strongest correlation with TKV. Multiple regression analysis revealed an independent correlation of KIM-1 with kidney volume, after adjustment for eGFR, osmolality, UACR, NGAL, UMOD, and CC16. In contrast, KIM-1 was not associated with renal function in multiple regression analysis adjusted for renal volume, osmolality, UACR, NGAL, KIM-1, UMOD, and CC16. KIM-1 expression was found in murine polycystic kidneys but not in wild type mice, driving the hypothesis that ADPKD patients may display higher urinary KIM-1 excretion [29]. KIM-1 has been identified as novel ciliary molecule. By interacting with the PKD2 Protein Transient Receptor Potential Polycystic 2, KIM-1 may be involved in cellular response to changes in extracellular fluid flow detected by the cilium [30]. To our knowledge urinary KIM-1 levels in ADPKD has only been reported once. In a study of Meijer et al increased KIM-1 levels in 24h urine samples of ADPKD patients were associated with total kidney volume, adjusted for age, gender and albuminuria compared with healthy volunteers [2]. In our study, KIM-1 was associated with TKV whereas renal function that is stable at early disease stage was not associated with KIM-1. Given the limited available data of KIM-1 in APDKD patients, it is not possible to draw a conclusion about the property of KIM-1 as biomarker in early ADPKD.

A defect in osmoregulation has been shown in animal models of ADPKD as well as in patients [31–33]. The impaired capacity of urine concentration is an early manifestation and can be observed in children [33,34]. With our study we confirm the independent association of osmolality and TKV as shown by Ho et al [33]. Following multiple adjustment, urine osmolality is independently associated with kidney volume and function in our cohort. Hence, the assessment of spot urine osmolality may add further information for disease state assessment in ADPKD patients at early disease stage.

Albuminuria is known as marker for kidney damage for years. Urinary albumin excretion is routinely assessed in the diagnosis of renal injury, due its urinary appearance prior to GFR decline in different renal diseases. Albuminuria is associated with CKD progression, decreasing eGFR, increasing TKV, myocardial infarction and mortality [24,35–39]. In our study, UACR predicts the variation in htTKV but did not qualify as predictor for kidney function.

KIM-1, urine osmolality and UACR are independently associated with disease state in our study, but no association of NGAL, UMOD and CC16 with kidney volume and function at early ADPKD state was found. NGAL has been extensively studied as biomarker in acute kidney injury, but only limited data is available reporting NGAL levels in ADPKD [7–11,40]. Boligano et al reported markedly increased urinary NGAL levels in ADPKD patients at late disease state (eGFR 59 ± 38 ml per minute, Cockcroft-Gault formula) compared with healthy volunteers [41]. Parikh and colleagues investigated serum and urinary NGAL levels over a three year period in participants of the CRISP study, with kidney function comparable to our investigated cohort (creatinine clearance >70 mls/min). The majority of subjects showed normal to low baseline urinary NGAL levels. Even so the levels increased during the study period with a drop at 2^nd^ and third year follow up, no association of NGAL quartiles with kidney volume and function were seen. Furthermore they showed highly elevated NGAL levels in cystic fluid in ADPKD patients compared to urine and serum values of healthy and of patients with AKI. The discrepancy of NGAL levels in urine and in cyst fluid may be attributable to a missing communication between tubules and detaching cysts in ADPKD [40]. In summary, NGAL levels may increase only in advanced disease and NGAL is not suitable to predict outcome at early stage when renal function is maintained.

To our knowledge, UMOD and CC16 levels have not been reported in ADPKD so far. In our study, no association of UMOD and CC16 with renal function and kidney volume was observed. Decreasing levels of urinary UMOD, which is the most abundant protein in human urine, have been reported in various settings of CKD [12–15]. Since the absolute values for urinary UMOD in our cohort are comparable with the ones reported in various cohorts, one could speculate that UMOD excretion decline starts in later stage of ADPKD [42,43]. CC16 is secreted by bronchial Clara cells and, after filtration, reabsorbed by receptor-mediated endocytosis in the early segments of the proximal tubule [17]. Hence, all disorders associated with defective proximal tubule endocytosis lead to the urinary loss of CC16 [44]. The described lack of association of CC16 with kidney volume in contrast to KIM-1 probably reflects the functional segmentation of the proximal tubule, with endocytosis being particularly active in the S1-S2 segments whereas secretory pathways take place in the S3 segment [45].

Our cross-sectional study has to be interpreted in the context of the study setting. We report independent association between biomarkers and outcome of a relatively high number of young ADPKD patients. We are not able to conclude about causal relationships between urinary biomarkers and outcome parameter. The parameters were only investigated at a single time point and we are not able to infer whether or not biomarker excretion precedes decrease in renal function and increase in kidney volume. Furthermore, our results are based on a single centre in the absence of comparative groups of healthy volunteers or other CKD patients, making it impossible to assess the specificity of our findings for ADPKD. To partially account for the study limitations, we performed internal validation of our data set by bootstrapping. Despite adjustment for multiple confounders of our predictive models, we are not able to fully eliminate the potential for bias and confounding. We followed a robust and reliable statistical approach to investigate the diagnostic properties of different urinary biomarkers in a large cohort of ADPKD patients in early disease stage. Based on our results we hypothesize that osmolality, UACR and KIM-1 may have the property to assess disease state at early ADPKD disease course, whereas NGAL, UMOD and CC16 seem not to qualify as biomarkers. Further studies are necessary to define the biomarker properties of the investigated parameter to predict disease burden in ADPKD.
